# Automatically Explaining Machine Learning Predictions on Severe Chronic Obstructive Pulmonary Disease Exacerbations: Retrospective Cohort Study

**DOI:** 10.2196/33043

**Published:** 2022-02-25

**Authors:** Siyang Zeng, Mehrdad Arjomandi, Gang Luo

**Affiliations:** 1 Department of Biomedical Informatics and Medical Education University of Washington Seattle, WA United States; 2 Medical Service San Francisco Veterans Affairs Medical Center San Francisco, CA United States; 3 Department of Medicine University of California San Francisco, CA United States

**Keywords:** chronic obstructive pulmonary disease, forecasting, machine learning, patient care management

## Abstract

**Background:**

Chronic obstructive pulmonary disease (COPD) is a major cause of death and places a heavy burden on health care. To optimize the allocation of precious preventive care management resources and improve the outcomes for high-risk patients with COPD, we recently built the most accurate model to date to predict severe COPD exacerbations, which need inpatient stays or emergency department visits, in the following 12 months. Our model is a machine learning model. As is the case with most machine learning models, our model does not explain its predictions, forming a barrier for clinical use. Previously, we designed a method to automatically provide rule-type explanations for machine learning predictions and suggest tailored interventions with no loss of model performance. This method has been tested before for asthma outcome prediction but not for COPD outcome prediction.

**Objective:**

This study aims to assess the generalizability of our automatic explanation method for predicting severe COPD exacerbations.

**Methods:**

The patient cohort included all patients with COPD who visited the University of Washington Medicine facilities between 2011 and 2019. In a secondary analysis of 43,576 data instances, we used our formerly developed automatic explanation method to automatically explain our model’s predictions and suggest tailored interventions.

**Results:**

Our method explained the predictions for 97.1% (100/103) of the patients with COPD whom our model correctly predicted to have severe COPD exacerbations in the following 12 months and the predictions for 73.6% (134/182) of the patients with COPD who had ≥1 severe COPD exacerbation in the following 12 months.

**Conclusions:**

Our automatic explanation method worked well for predicting severe COPD exacerbations. After further improving our method, we hope to use it to facilitate future clinical use of our model.

**International Registered Report Identifier (IRRID):**

RR2-10.2196/13783

## Introduction

### Background

Chronic obstructive pulmonary disease (COPD) is a leading cause of death [1] and affects 6.5% of American adults [2]. In the United States, COPD leads to 0.7 million inpatient stays and 1.5 million emergency department (ED) visits every year [2]. Severe COPD exacerbations are exacerbations that need inpatient stays or ED visits [3]. These exacerbations often result in irreversible deterioration in health status and lung function [4-9] and account for 90.3% of the US $32.1 billion total annual medical costs of the United States associated with COPD [2,10]. Many of these exacerbations, which include 47% of inpatient stays and many ED visits because of COPD, are regarded as preventable with suitable outpatient care [3,11]. To reduce severe COPD exacerbations, many health care systems and health plans use predictive models to identify high-risk patients [12] for preventive care management [13]. Once a patient is enrolled in the care management program, care managers will regularly follow up with the patient on the phone to assess the patient’s health status and help schedule health and related services. For patients with COPD, successful care management can cut up to 40% of their inpatient stays [14] and 27% of their ED visits [15].

As a care management program can take ≤3% of patients because of resource limits [16], the effectiveness of the program depends critically on the performance of the predictive model that is used. To optimize the allocation of precious care management resources and improve the outcomes for high-risk patients with COPD, we recently built the most accurate model to date to predict severe COPD exacerbations in the following 12 months [17]. Our model achieved an area under the receiver operating characteristic curve of 0.866, a sensitivity of 56.6% (103/182), and a specificity of 91.17% (6698/7347). In comparison, to the best of our knowledge, each published prior model for this prediction target [18-51] had an area under the receiver operating characteristic curve ≤0.809 and a sensitivity <50% when the specificity was set at approximately 91%. Our model is based on the machine learning algorithm of extreme gradient boosting (XGBoost) [52]. As is the case with most machine learning models, our model does not explain its predictions, forming a barrier for clinical use [53]. Offering explanations is essential for care managers to make sense of and trust the model’s predictions to make care management enrollment decisions and identify suitable interventions. Currently, there is no consensus on what explanation means for machine learning predictions. In this paper, by explaining the prediction that a machine learning model makes on a patient, we mean to find ≥1 rule whose left-hand side is fulfilled by the patient and whose right-hand side is consistent with the prediction. Previously, we developed a method to automatically provide rule-type explanations for any machine learning model’s predictions on tabular data and suggest tailored interventions with no loss of model performance [54-58]. This method has been tested before for asthma outcome prediction but not for COPD outcome prediction.

### Objective

The goal of this particular study is to assess the generalizability of our automatic explanation method for predicting severe COPD exacerbations. After further improving our method in the future, our eventual goal is that care managers can use our method to make COPD care management enrollment and intervention decisions more quickly and reliably.

## Methods

### Ethics Approval and Study Design

The institutional review board of the University of Washington Medicine (UWM) approved this retrospective cohort study (STUDY00000118) using administrative and clinical data.

### Patient Population

In Washington state, the UWM is the largest academic health care system. The enterprise data warehouse of the UWM contains administrative and clinical data from 12 clinics and 3 hospitals. This study used the same patient cohort as our previous predictive modeling study [17]. The patient cohort included all patients with COPD who visited the UWM facilities between 2011 and 2019. As adapted from the literature [59-62], a patient was deemed to have COPD if the patient was aged at least 40 years and met at least one of the following criteria:

1. The patient had “an outpatient visit diagnosis code of COPD (International Classification of Diseases, Ninth Revision (ICD-9): 491.22, 491.21, 491.9, 491.8, 493.2x, 492.8, 496; International Classification of Diseases, Tenth Revision (ICD-10): J42, J41.8, J44.*, J43.*) followed by ≥1 prescription of long-acting muscarinic antagonist (aclidinium, glycopyrrolate, tiotropium, and umeclidinium) within 6 months”
2. The patient had “≥1 ED or ≥2 outpatient visit diagnosis codes of COPD (International Classification of Diseases, Ninth Revision: 491.22, 491.21, 491.9, 491.8, 493.2x, 492.8, 496; International Classification of Diseases, Tenth Revision: J42, J41.8, J44.*, J43.*)”
3. The patient had “≥1 inpatient stay discharge having a principal diagnosis code of COPD (International Classification of Diseases, Ninth Revision: 491.22, 491.21, 491.9, 491.8, 493.2x, 492.8, 496; International Classification of Diseases, Tenth Revision: J42, J41.8, J44.*, J43.*)”
4. The patient had “≥1 inpatient stay discharge having a principal diagnosis code of respiratory failure (International Classification of Diseases, Ninth Revision: 518.82, 518.81, 799.1, 518.84; International Classification of Diseases, Tenth Revision: J96.0*, J80, J96.9*, J96.2*, R09.2) and a secondary diagnosis code of acute COPD exacerbation (International Classification of Diseases, Ninth Revision: 491.22, 491.21, 493.22, 493.21; International Classification of Diseases, Tenth Revision: J44.1, J44.0)” [17].

We used one exclusion criterion: when calculating the data instances in a given year, the patients who died or had no encounter at the UWM during that year were excluded.

### Data Set

This study used the same structured data set as our previous predictive model paper [17]. The data set contained the administrative and clinical data of the patient cohort’s encounters at the 12 UWM clinics and 3 UWM hospitals between 2011 and 2020.

### Prediction Target (Dependent or Outcome Variable)

This study used the same prediction target as our previous predictive model [17]. For a patient with COPD and ≥1 encounter at the UWM in a particular year (index year), we used patient data up to the end of the year to predict the outcome—whether the patient would have ≥1 severe COPD exacerbation in the following 12 months. A severe COPD exacerbation is defined as an inpatient stay or an ED visit with a principal diagnosis of COPD (International Classification of Diseases, Ninth Revision: 491.22, 491.21, 491.9, 491.8, 493.2x, 492.8, 496; International Classification of Diseases, Tenth Revision: J42, J41.8, J44.*, J43.*).

### Data Preprocessing, Predictive Model, and Features (Independent Variables)

We applied the same methods as in our previous predictive model paper [17] to perform data preprocessing. Using the upper and lower bounds provided by a clinical expert in our team, as well as the upper and lower bounds from the Guinness World Records, we pinpointed the biologically implausible values, marked them missing, and normalized each numerical feature. Our model used 229 features and the XGBoost classification algorithm [52] to make predictions. As listed in the second table in the web-based multimedia appendix of our previous paper [17], these features were calculated on the attributes in our structured data set and covered various aspects such as vital signs, diagnoses, visits, procedures, medications, laboratory tests, and patient demographics. An example feature is the number of days since the patient had the last diagnosis of acute COPD exacerbation. Each input data instance to the predictive model contained these 229 features, corresponded to a distinct patient and index year pair, and was used to predict the outcome of the patient in the following 12 months. As in our previous predictive model paper [17], the cutoff threshold for binary classification was set at the top 10% of patients with the largest predicted risk. A care management program can take ≤3% of patients because of resource limits [16]. After using our model to identify the top 10% of patients with the largest predicted risk and using our automatic explanation method to explain the predictions, care managers could review patient charts, consider factors such as social dimensions, and choose ≤3% of patients for care management enrollment. A value of 10% was chosen to strike a balance between covering a large percentage of patients who would have ≥1 severe COPD exacerbation in the following 12 months and keeping the care managers’ workload manageable.

### Review of Our Automatic Explanation Method

#### Overview

Previously, we developed a method to automatically provide rule-type explanations for any machine learning model’s predictions on tabular data and suggest tailored interventions with no loss of model performance [54-58]. When creating the automatic explanation function before the prediction time, our method requires ≥1 expert in the function’s design team to manually provide some information, such as marking the feature–value pairs that could have a positive correlation with the bad outcome value and compiling interventions for these feature–value pair items. This can typically be performed in a few man-hours. Once this information is obtained and stored in the function’s knowledge base, our method can automatically explain the machine learning model’s predictions and suggest tailored interventions at the prediction time.

#### Main Idea

Our automatic explanation method [54-58] uses 2 models at the same time to separate making predictions and providing explanations. Each model plays a different role. The first model is used to predict the outcome. This model can be any model that takes continuous and categorical features as its inputs and is typically chosen to be the model that performs the best at making predictions. The second model comprises class-based association rules [63,64] mined from the training set. We use the second model to explain the first model’s predictions rather than to make predictions. After we convert each continuous feature into ≥1 categorical feature via automatic discretization [63,65], the association rules are mined using the Apriori algorithm, whereas other standard methods such as frequent pattern growth can also be used [64]. Every rule shows that a feature pattern links to a value *z* of the outcome variable in the form of:

*p_1_* AND *p_2_* AND...AND *p_k_*→*z*. **(1)**

Here, each item *p_i_* (1≤*i*≤*k*) is a feature-value pair (*x*, *c*), indicating that feature *x* has a value *c* if *c* is a value or a value within *c* if *c* is a range. The values of *k* and *z* can vary by rules. For the binary classification of good versus bad outcomes, *z* is usually the bad outcome value. The rule indicates that a patient’s outcome tends to take the value *z* if the patient satisfies all of *p_1_*, *p_2_*,..., and *p_k_*. The following is an example of a rule:

The patient’s last diagnosis of acute COPD exacerbation was from the past 81.4 days AND the patient’s COPD reliever prescriptions in the past year included >10 distinct medications → The patient will probably have at least one severe COPD exacerbation in the following 12 months.

#### Mining and Pruning Rules

Each rule has two quality measures: commonality and confidence. For a rule:

*p_1_* AND *p_2_* AND...AND *p_k_*→*z*, **(1)**

its commonality is defined as the percentage of data instances satisfying *p_1_*, *p_2_*,..., and *p_k_* among all the data instances linked to *z*. Its confidence is defined as the percentage of data instances linked to *z* among all the data instances satisfying *p_1_*, *p_2_*,..., and *p_k_*. Commonality measures the coverage of a rule within the context of *z*. Confidence measures the precision of a rule.

The process of mining and pruning rules is controlled by five parameters: the number of top features that are used to form rules, upper limit of the number of items on the left-hand side of a rule, lower limit of confidence, lower limit of commonality, and upper limit of the confidence difference. Our method uses rules that each contains at most the upper limit number of items on its left-hand side, has a commonality that is greater than or equal to the lower limit of commonality, and has a confidence that is greater than or equal to the lower limit of confidence.

Our automatic explanation method is intended to be used for real-time clinical decision support. Once the first model provides its predicted outcome of a patient, we need to use the second model to provide automatic explanations for the prediction quickly, ideally within a subsecond. For this purpose, we need to control the number of association rules in the second model to help reduce the overhead of retrieving and ranking the relevant rules at the prediction time. We used the following three techniques to cut the number of rules:

1. Some machine learning algorithms, such as XGBoost [52], automatically calculate the importance value of each feature. When the data set included many features, we used only the top few features in the first model with the highest importance values to form rules. Usually, we set the number of top features to be used to the maximum possible number without making the association rule mining process run out of memory.
2. A rule *r_1_* was dropped if there exists another rule *r_2_* satisfying three conditions: *r_1_* and *r_2_* have the same value on their right-hand sides; the items on the left-hand side of *r_2_* are a proper subset of the items on the left-hand side of *r_1_* (ie, *r_2_* is more general than *r_1_*); and the confidence of *r_2_* is greater than or equal to the confidence of *r_1_*− the upper limit of the confidence difference.
3. All distinct feature–value pairs were examined and labeled by a clinical expert in the automatic explanation function’s design team. When forming rules, we used only those feature–value pairs that the clinical expert deemed could have a positive correlation with the bad outcome value.

For every feature-value pair item used to form association rules, a clinical expert in the automatic explanation function’s design team compiled ≥0 intervention. An item is termed actionable if it is associated with ≥1 intervention. These interventions are automatically attached to the rules whose left-hand sides contain this item. A rule is termed actionable if its left-hand side contains ≥1 actionable item and, in turn, is associated with ≥1 intervention. In theory, for each combination of feature–value pair items that appears on the left-hand side of ≥1 mined rule, the clinical expert could compile additional interventions to be automatically attached to the rules whose left-hand sides contain this combination if these interventions have not already been compiled for any individual feature–value pair item in the combination. In practice, we have not needed to do this for predicting severe COPD exacerbations, whereas such a need could occur in some other clinical prediction tasks in the future.

#### Explaining the Predictions

For each patient predicted by the first model to have a bad outcome, we explained the prediction by presenting the association rules in the second model whose left-hand sides are fulfilled by the patient and whose right-hand sides have the bad outcome value. The rules were sorted using the method given in our paper [57]. This method incorporates 5 factors into a rule-scoring function, striking a balance among them. These factors include confidence, commonality, number of items on the left-hand side of the rule, whether the rule is actionable, and the degree of information redundancy with the higher-ranked rules. The rules are ranked based on the computed scores in an iterative fashion. Every rule offers an explanation for why the patient is predicted to have a bad outcome. For each actionable rule that is presented, the associated interventions are shown next to it. This helps the user of the automatic explanation function pinpoint suitable interventions for the patient. Typically, the rules in the second model provide common reasons for a patient to have a bad outcome. Although some patients could have bad outcomes because of rare reasons not covered by these rules, the second model usually explains most, although not all, of the bad outcomes correctly predicted by the first model.

### Parameter Setting

Our model [17] used 229 features to predict patient outcomes. In this study, we used the top 80 features that our model ranked with the highest importance values to form association rules. Regardless of whether all 229 features or only the top 80 features were used, our model had the same area under the receiver operating characteristic curve of 0.866.

As in our prior study on automatically explaining predictions of asthma outcomes on the UWM data [55], we set the upper limit of the number of items on the left-hand side of a rule to 5, the lower limit of commonality to 1%, and the lower limit of confidence to 50%. The last 2 values were commonly used to mine association rules [63], whereas commonality was essentially support computed on all the data instances linked to the bad outcome [54]. The first value struck a balance between the explanation power of our automatic explanation method and not making the rules too complex to understand. To set the upper limit value of the confidence difference, we plotted the number of association rules remaining from the rule pruning process versus the upper limit of the confidence difference. Our prior automatic explanation papers [54-56,58] showed that the number of remaining rules first decreased rapidly as the upper limit of the confidence difference increased and then slowly decreased after the upper limit of the confidence difference became large enough. The upper limit value of the confidence difference was set at a point where a further increase in the confidence difference had a minor impact on reducing the number of remaining rules.

### Data Analysis

#### Split of the Training and Test Sets

We adopted the method from our previous predictive model paper [17] to split the entire data set into the training and test sets. As the outcomes were from the following year, the data set contained 9 years of effective data (2011-2019) over the 10-year period of 2011 to 2020. To reflect how our predictive model and our automatic explanation method will be used in clinical practice in the future, we used the 2011 to 2018 data as the training set to train our model and compute the association rules used by our automatic explanation method and the 2019 data as the test set to assess the performance of our model and our automatic explanation method.

#### Providing Examples of Automatic Explanations

To give the reader a concrete feeling of the results produced by our automatic explanation method, we randomly selected 3 example patients from the patients who were correctly predicted by our model to have ≥1 severe COPD exacerbation in the following 12 months and for whom our automatic explanation method could offer ≥1 explanation. For each example patient, we listed the top 3 explanations given by our automatic explanation method.

#### Performance Metrics

We examined the performance of our automatic explanation method using the following performance metrics from our prior automatic explanation papers [54-56,58]. Regarding the explanation power of our automatic explanation method, a performance metric is the percentage of patients for whom our method could provide explanations among the patients with COPD who were correctly predicted by our model to have ≥1 severe COPD exacerbation in the following 12 months. We assessed both the average and median number of (actionable) rules matching such a patient. A rule matches a patient if the patient satisfies all items on its left-hand side.

As shown by our prior automatic explanation papers [54-56,58], many rules matching a patient often differ from each other by only 1 item on their left-hand sides. In this case, the number of rules greatly exceeded the amount of nonrepeated information contained in these rules. To provide a comprehensive overview of the amount of information provided by the automatic explanations, we examined the distributions of (1) the number of (actionable) rules and (2) the number of unique actionable items in the rules matching a patient who was correctly predicted by our model to have ≥1 severe COPD exacerbation in the following 12 months.

## Results

### Characteristics of Our Patient Cohort

Each data instance corresponds to a distinct patient and index year pair. Tables 1 and 2 summarize the patient demographic and clinical characteristics of the data instances in the training and test sets, respectively. These 2 sets of characteristics were relatively similar to each other. In the training set, 5.66% (2040/36,047) of the data instances were related to severe COPD exacerbations in the following 12 months. In the test set, 2.42% (182/7529) of the data instances were related to severe COPD exacerbations in the following 12 months. A detailed comparison of these 2 sets of characteristics was provided in our previous predictive model paper [17].

**Table 1:** The patient demographic and clinical characteristics of the data instances in the training set.

Patient characteristics			Data instances related to no severe COPD^a^ exacerbation in the following 12 months (n=34,007), n (%)		Data instances related to severe COPD exacerbations in the following 12 months (n=2040), n (%)		Data instances (n=36,047), n (%)
**Sex**							
	Female	14,665 (43.12)		749 (36.72)		15,414 (42.76)	
	Male	19,342 (56.88)		1291 (63.28)		20,633 (57.24)	
**Age (years)**							
	40-65	17,574 (51.68)		1219 (59.75)		18,793 (52.13)	
	>65	16,433 (48.32)		821 (40.25)		17,254 (47.87)	
**Race**							
	White	26,117 (76.8)		1330 (65.2)		27,447 (76.14)	
	Black or African American	4271 (12.56)		524 (25.69)		4795 (13.3)	
	Asian	1948 (5.73)		144 (7.06)		2092 (5.8)	
	American Indian or Alaska Native	687 (2.02)		26 (1.27)		713 (1.98)	
	Native Hawaiian or other Pacific Islander	176 (0.52)		8 (0.39)		184 (0.51)	
	Other, unknown, or not reported	808 (2.37)		8 (0.39)		816 (2.27)	
**Ethnicity**							
	Hispanic	804 (2.36)		53 (2.6)		857 (2.38)	
	Non-Hispanic	30,644 (90.11)		1941 (95.15)		32,585 (90.39)	
	Unknown or not reported	2559 (7.53)		46 (2.25)		2605 (7.23)	
**Insurance**							
	Public	27,831 (81.84)		1767 (86.62)		29,598 (82.11)	
	Private	16,679 (49.05)		834 (40.88)		17,513 (48.58)	
	Self-paid or charity	1765 (5.19)		229 (11.23)		1994 (5.53)	
**Number of years since the first encounter related to COPD in the data set**							
	≤3	28,749 (84.54)		1566 (76.76)		30,315 (84.1)	
	>3	5258 (15.46)		474 (23.24)		5732 (15.90)	
**Smoking status**							
	Current smoker	15,863 (46.65)		1089 (53.38)		16,952 (47.03)	
	Former smoker	7022 (20.65)		345 (16.91)		7367 (20.44)	
	Never smoker or unknown	11,122 (32.7)		606 (29.71)		11,728 (32.53)	
**COPD medication prescription**							
	SABA^b^	20,865 (61.36)		1684 (82.55)		22,549 (62.55)	
	SAMA^c^	8566 (25.19)		1042 (51.08)		9608 (26.65)	
	SABA and SAMA combination	6364 (18.71)		810 (39.71)		7174 (19.9)	
	LABA^d^	8062 (23.71)		842 (41.27)		8904 (24.7)	
	LAMA^e^	9242 (27.18)		1001 (49.07)		10,243 (28.42)	
	LABA and LAMA combination	386 (1.14)		40 (1.96)		426 (1.18)	
	ICS^f^	12,208 (35.9)		1119 (54.85)		13,327 (36.97)	
	ICS and LABA combination	7544 (22.18)		782 (38.33)		8326 (23.1)	
	ICS, LABA, and LAMA combination	16 (0.05)		0 (0)		16 (0.04)	
	Systemic corticosteroid	10,149 (29.84)		1144 (56.08)		11,293 (31.33)	
	Phosphodiesterase-4 inhibitor	84 (0.25)		10 (0.49)		94 (0.26)	
**Comorbidity**							
	Anxiety or depression	10,061 (29.59)		725 (35.54)		10,786 (29.92)	
	Allergic rhinitis	2271 (6.68)		174 (8.53)		2445 (6.78)	
	Asthma	4377 (12.87)		417 (20.44)		4794 (13.3)	
	Diabetes	7177 (21.1)		446 (21.86)		7623 (21.15)	
	Congestive heart failure	5568 (16.37)		495 (24.26)		6063 (16.82)	
	Eczema	1460 (4.29)		98 (4.8)		1558 (4.32)	
	Hypertension	17,211 (50.61)		1150 (56.37)		18,361 (50.94)	
	Gastroesophageal reflux	6655 (19.57)		507 (24.85)		7162 (19.87)	
	Ischemic heart disease	6934 (20.39)		486 (23.82)		7420 (20.58)	
	Obesity	3232 (9.5)		255 (12.5)		3487 (9.67)	
	Lung cancer	742 (2.18)		52 (2.55)		794 (2.2)	
	Sleep apnea	2926 (8.6)		253 (12.4)		3179 (8.82)	
	Sinusitis	1299 (3.82)		83 (4.07)		1382 (3.83)	

^a^COPD: chronic obstructive pulmonary disease.

^b^SABA: short-acting beta-2 agonist.

^c^SAMA: short-acting muscarinic antagonist.

^d^LABA: long-acting beta-2 agonist.

^e^LAMA: long-acting muscarinic antagonist.

^f^ICS: inhaled corticosteroid.

**Table 2:** The patient demographic and clinical characteristics of the data instances in the test set.

Patient characteristics		Data instances related to no severe COPD^a^ exacerbation in the following 12 months (n=7347), n (%)	Data instances related to severe COPD exacerbations in the following 12 months (n=182), n (%)	Data instances (n=7529), n (%)
**Sex**				
	Female	3242 (44.13)	47 (25.8)	3289 (43.68)
	Male	4105 (55.87)	135 (74.2)	4240 (56.32)
**Age (years)**				
	40-65	3324 (45.24)	118 (64.8)	3442 (45.72)
	>65	4023 (54.76)	64 (35.2)	4087 (54.28)
**Race**				
	White	5682 (77.34)	111 (61.0)	5793 (76.94)
	Black or African American	839 (11.42)	57 (31.3)	896 (11.9)
	Asian	432 (5.88)	7 (3.9)	439 (5.83)
	American Indian or Alaska Native	151 (2.06)	5 (2.7)	156 (2.07)
	Native Hawaiian or other Pacific Islander	51 (0.69)	2 (1.1)	53 (0.71)
	Other, unknown, or not reported	192 (2.61)	0 (0.0)	192 (2.55)
**Ethnicity**				
	Hispanic	185 (2.52)	3 (1.6)	188 (2.5)
	Non-Hispanic	6909 (94.04)	179 (98.4)	7088 (94.14)
	Unknown or not reported	253 (3.44)	0 (0)	253 (3.36)
**Insurance**				
	Public	6722 (91.49)	179 (98.4)	6901 (91.66)
	Private	4532 (61.69)	110 (60.4)	4642 (61.65)
	Self-paid or charity	499 (6.79)	41 (22.5)	540 (7.17)
**Number of years since the first encounter related to COPD in the data set**				
	≤3	5073 (69.05)	81 (44.5)	5154 (68.46)
	>3	2274 (30.95)	101 (55.5)	2375 (31.54)
**Smoking status**				
	Current smoker	3781 (51.46)	112 (61.5)	3893 (51.71)
	Former smoker	1242 (16.91)	25 (13.7)	1267 (16.83)
	Never smoker or unknown	2324 (31.63)	45 (24.7)	2369 (31.47)
**COPD medication prescription**				
	SABA^b^	4083 (55.57)	158 (86.8)	4241 (56.33)
	SAMA^c^	1134 (15.43)	68 (37.4)	1202 (15.96)
	SABA and SAMA combination	1694 (23.06)	115 (63.2)	1809 (24.03)
	LABA^d^	1683 (22.91)	77 (42.3)	1760 (23.38)
	LAMA^e^	1951 (26.56)	110 (60.4)	2061 (27.37)
	LABA and LAMA combination	388 (5.28)	12 (6.6)	400 (5.31)
	ICS^f^	2537 (34.53)	98 (53.8)	2635 (35)
	ICS and LABA combination	1729 (23.53)	75 (41.2)	1804 (23.96)
	ICS, LABA, and LAMA combination	68 (0.93)	1 (0.5)	69 (0.92)
	Systemic corticosteroid	2282 (31.06)	103 (56.6)	2385 (31.68)
	Phosphodiesterase-4 inhibitor	24 (0.33)	2 (1.1)	26 (0.35)
**Comorbidity**				
	Anxiety or depression	2090 (28.45)	63 (34.6)	2153 (28.6)
	Allergic rhinitis	396 (5.39)	14 (7.7)	410 (5.45)
	Asthma	1053 (14.33)	43 (23.6)	1096 (14.56)
	Diabetes	1649 (22.44)	40 (22)	1689 (22.43)
	Congestive heart failure	1369 (18.63)	43 (23.6)	1412 (18.75)
	Eczema	247 (3.36)	11 (6)	258 (3.43)
	Hypertension	3686 (50.17)	105 (57.7)	3791 (50.35)
	Gastroesophageal reflux	1396 (19)	47 (25.8)	1443 (19.17)
	Ischemic heart disease	1604 (21.83)	54 (29.7)	1658 (22.02)
	Obesity	648 (8.82)	21 (11.5)	669 (8.89)
	Lung cancer	200 (2.72)	3 (1.6)	203 (2.7)
	Sleep apnea	887 (12.07)	28 (15.4)	915 (12.15)
	Sinusitis	272 (3.7)	7 (3.8)	279 (3.71)

^a^COPD: chronic obstructive pulmonary disease.

^b^SABA: short-acting beta-2 agonist.

^c^SAMA: short-acting muscarinic antagonist.

^d^LABA: long-acting beta-2 agonist.

^e^LAMA: long-acting muscarinic antagonist.

^f^ICS: inhaled corticosteroid.

### The Number of Association Rules

**Figure 1:**
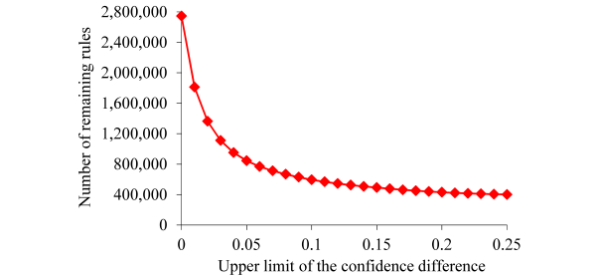
The number of remaining association rules versus the upper limit of the confidence difference.

Using the top 80 features ranked with the highest importance values in our predictive model, 7,729,134 association rules were mined from the training set. Figure 1 shows the number of remaining rules versus the upper limit of the confidence difference. The number of remaining rules first rapidly decreased as the upper limit of the confidence difference increased and then slowly decreased after the upper limit of the confidence difference became ≥0.15. We set the upper limit of the confidence difference to the value of 0.15, resulting in 492,803 remaining rules.

The top 80 features totally had 219 distinct feature–value pairs, 141 (64.4%) of which were actionable. A clinical expert on COPD (MA) in our team reviewed all distinct feature–value pairs of the top 80 features and labeled those that could have a positive correlation with severe COPD exacerbations in the following 12 months. After dropping the rules containing any other feature–value pair items, 460,592 rules were left. These rules were all actionable.

### Examples of the Produced Automatic Explanations

To give the reader a concrete feeling of the results produced by our automatic explanation method, we randomly selected 3 example patients from the patients who were correctly predicted by our model to have ≥1 severe COPD exacerbation in the following 12 months and for whom our automatic explanation method could offer ≥1 explanation. Tables 3-5 show the top 3 explanations that our automatic explanation method provided for every example patient.

**Table 3:** The top 3 association rules generated for the first example patient.

Rank, rule, and item on the rule’s left-hand side		Interpretation of the item	Interventions linked to the item
**Rank 1: The patient’s last diagnosis of acute COPD^a^ exacerbation was from the past 81.4 days AND the patient’s COPD reliever prescriptions in the past year included >10 distinct medications** **→** **the patient will probably have at least one severe COPD exacerbation in the following 12 months**			
	The patient’s last diagnosis of acute COPD exacerbation was from the past 81.4 days	Having a recent acute COPD exacerbation shows a need for better control of the disease.	Provide education on managing COPD and more frequent follow-ups Ensure use of appropriate COPD medications Consider influenza shot, pneumonia vaccination, or smoking cessation Assess the need for pulmonary rehabilitation or home care Ensure that the patient has a primary care provider or is referred to a specialist
	The patient’s COPD reliever prescriptions in the past year included >10 distinct medications	Using many rescue medications for COPD indicates ineffective regimen, poor treatment adherence, or poor control of the disease.	Simplify COPD medications to once-a-day formulations or combination medications Address concerns for adverse interactions between medications Provide education on the correct use of COPD medications or inhalers Consider strategies to improve medication adherence such as providing reminders for taking medications in time Medication reconciliation review by a physician or a pharmacist
**Rank 2: The patient had between 8 and 19 diagnoses of acute COPD exacerbation in the past year AND the patient’s last COPD diagnosis was from the past 25.6 days AND the patient’s nebulizer medication prescriptions in the past year included >11 medications** **→** **the patient will probably have at least one severe COPD exacerbation in the following 12 months**			
	The patient had between 8 and 19 diagnoses of acute COPD exacerbation in the past year	Frequently having acute COPD exacerbations shows a need for better control of the disease.	Provide education on managing COPD and more frequent follow-ups Ensure use of appropriate COPD medications Consider influenza shot, pneumonia vaccination, or smoking cessation Assess the need for pulmonary rehabilitation or home care
	The patient’s last COPD diagnosis was from the past 25.6 days	Having a recent COPD diagnosis associated with an ED^b^ visit or an inpatient stay indicates poor control of the disease.	Provide education on managing COPD and more frequent follow-ups Ensure use of appropriate COPD medications Consider influenza shot, pneumonia vaccination, or smoking cessation Assess the need for pulmonary rehabilitation or home care
	The patient’s nebulizer medication prescriptions in the past year included >11 medications	Using many medications for COPD with a nebulizer indicates an ineffective regimen, poor treatment adherence, or poor control of the disease. Using nebulizer medications could be a sign of having a mild exacerbation or more severe COPD.	Simplify COPD medications to once-a-day formulations or combination medications Address concerns for adverse interactions between medications Provide education on the correct use of COPD medications or inhalers Consider strategies to improve medication adherence such as providing reminders for taking medications in time Medication reconciliation review by a physician or a pharmacist
**Rank 3: The** **patient’s** **average length of an inpatient stay in the past year was between 0.61 and 7.66 days AND the patient’s last outpatient visit on COPD occurred in the past 82.4 days AND the patient’s nebulizer medication prescriptions in the past year included >11 medications AND the patient’s maximum percentage of neutrophils in the past year was >76.5%** **→** **the patient will probably have at least one severe COPD exacerbation in the following 12 months**			
	The patient’s average length of an inpatient stay in the past year was between 0.61 and 7.66 days	Having a long inpatient stay can indicate that the patient has a more severe disease or comorbidities.	Ensure that the patient has a primary care provider Assess the need for home care or referral to a skilled nursing facility Provide education on managing COPD and resources for care Ensure use of appropriate COPD medications
	The patient’s last outpatient visit on COPD occurred in the past 82.4 days	If the patient’s last outpatient visit on COPD was for acute problems with COPD, it could indicate poor control of the disease and a need for additional support to control COPD.	Provide education on managing COPD and resources for care Ensure use of appropriate COPD medications Assess the need for home care or pulmonary rehabilitation
	The patient’s nebulizer medication prescriptions in the past year included >11 medications	Using many medications for COPD with a nebulizer indicates an ineffective regimen, poor treatment adherence, or poor control of the disease. Using nebulizer medications could be a sign of having a mild exacerbation or more severe COPD.	Simplify COPD medications to once-a-day formulations or combination medications Address concerns for adverse interactions between medications Provide education on the correct use of COPD medications or inhalers Consider strategies to improve medication adherence such as providing reminders for taking medications in time Medication reconciliation review by a physician or a pharmacist
	The patient’s maximum percentage of neutrophils in the past year was >76.5%	Having a large percentage of neutrophils can indicate infections or distress.	Evaluate the respiratory system, for example, using radiographic imaging Consider doing diagnostic tests such as viral panel, sputum culture, or procalcitonin Evaluate other potential morbidities such as cardiovascular disease with an electrocardiogram, echocardiography, or laboratory tests such as brain natriuretic peptide or D-dimer

^a^COPD: chronic obstructive pulmonary disease.

^b^ED: emergency department.

**Table 4:** The top 3 association rules generated for the second example patient.

Rank, rule, and item on the rule’s left-hand side				Interpretation of the item		Interventions linked to the item
**Rank 1: The patient’s last diagnosis of acute COPD^a^ exacerbation was from the past 81.4 days AND the patient had >2 ED^b^ visits in the past 6 months AND the patient’s nebulizer medication prescriptions in the past year included >11 medications** **→** **the patient will probably have at least one severe COPD exacerbation in the following 12 months**						
	The patient’s last diagnosis of acute COPD exacerbation was from the past 81.4 days		Having a recent acute COPD exacerbation shows a need for better control of the disease.		Provide education on managing COPD and more frequent follow-ups Ensure use of appropriate COPD medications Consider influenza shot, pneumonia vaccination, or smoking cessation Assess the need for pulmonary rehabilitation or home care Ensure that the patient has a primary care provider or is referred to a specialist	
	The patient had >2 ED visits in the past 6 months		Using the ED indicates poor control of conditions or a lack of access to primary, specialty, or home care.		Provide education on managing COPD and more frequent follow-ups Ensure use of appropriate COPD medications Consider influenza shot, pneumonia vaccination, or smoking cessation Assess the need for pulmonary rehabilitation or home care Ensure that the patient has a primary care provider or is referred to a specialist	
	The patient’s nebulizer medication prescriptions in the past year included >11 medications		Using many medications for COPD with a nebulizer indicates an ineffective regimen, poor treatment adherence, or poor control of the disease. Using nebulizer medications could be a sign of having a mild exacerbation or more severe COPD.		Simplify COPD medications to once-a-day formulations or combination medications Address concerns for adverse interactions between medications Provide education on the correct use of COPD medications or inhalers Consider strategies to improve medication adherence such as providing reminders for taking medications in time Medication reconciliation review by a physician or a pharmacist	
**Rank 2:** **The patient’s maximum BMI in the past year was <22.81 AND the patient’s last ED visit related to COPD occurred no less than 27.2 days ago and no more than 94.3 days ago AND the patient’s average length of stay of an ED visit in the past year was between 0.03 and 0.29 day AND the patient had between 2 and 4 encounters related to acute COPD exacerbation or respiratory failure in the past year→ the patient will probably have at least one severe COPD exacerbation in the following 12 months**						
	The patient’s maximum BMI in the past year was <22.81		Having an unintentional weight loss can indicate comorbidities or other complications, such as malnutrition or metabolic syndrome.		Optimize nutritional status to address low BMI Provide dietary education and advise appropriate exercise	
	The patient’s last ED visit related to COPD occurred no less than 27.2 days ago and no more than 94.3 days ago		Having a recent ED visit related to COPD shows a need for better control of the disease.		Provide education on managing COPD and more frequent follow-ups Ensure use of appropriate COPD medications Consider influenza shot, pneumonia vaccination, or smoking cessation Assess the need for pulmonary rehabilitation or home care Ensure that the patient has a primary care provider or is referred to a specialist	
	The patient’s average length of stay of an ED visit in the past year was between 0.03 and 0.29 day		Using the ED indicates poor control of conditions or a lack of access to primary, specialty, or home care.		Provide education on managing COPD and more frequent follow-ups Ensure use of appropriate COPD medications Consider influenza shot, pneumonia vaccination, or smoking cessation Assess the need for pulmonary rehabilitation or home care Ensure that the patient has a primary care provider or is referred to a specialist	
	The patient had between 2 and 4 encounters related to acute COPD exacerbation or respiratory failure in the past year		Frequently having acute COPD exacerbations or respiratory failures shows a need for better control of the disease.		Provide education on managing COPD and more frequent follow-ups Ensure use of appropriate COPD medications Consider influenza shot, pneumonia vaccination, or smoking cessation Assess the need for pulmonary rehabilitation or home care Ensure that the patient has a primary care provider or is referred to a specialist	
**Rank 3: The patient had between 3 and 5 ED visits in the past year AND the patient’s minimum SpO_2_^c^ in the past year was between 17% and 89.5% AND the patient’s maximum percentage of neutrophils in the past year was >76.5% AND the patient smoked >0.48 pack of cigarettes per day in the past year** **→** **the patient will probably have at least one severe COPD exacerbation in the following 12 months**						
		The patient had between 3 and 5 ED visits in the past year	Using the ED indicates poor control of conditions or a lack of access to primary, specialty, or home care.		Provide education on managing COPD and more frequent follow-ups Ensure use of appropriate COPD medications Consider influenza shot, pneumonia vaccination, or smoking cessation Assess the need for pulmonary rehabilitation or home care Ensure that the patient has a primary care provider or is referred to a specialist	
	The patient’s minimum SpO_2_ in the past year was between 17% and 89.5%		Having a low SpO_2_ indicates worsening of symptoms or other complications such as hypoxemia.		Evaluate for cardiopulmonary causes of hypoxemia Consider nighttime oximetry or sleep study to evaluate for nighttime hypoxemia or sleep apnea Assess the need for home oxygen or nighttime noninvasive ventilation	
	The patient’s maximum percentage of neutrophils in the past year was >76.5%		Having a large percentage of neutrophils can indicate infections or distress.		Evaluate the respiratory system, for example, using radiographic imaging Consider doing diagnostic tests such as viral panel, sputum culture, or procalcitonin Evaluate other potential morbidities such as cardiovascular disease with an electrocardiogram, echocardiography, or laboratory tests such as brain natriuretic peptide or D-dimer	
	The patient smoked >0.48 pack of cigarettes per day in the past year		Smoking is a key risk factor for COPD complications.		Provide education on the health risks of smoking Suggest and provide support for smoking cessation	

^a^COPD: chronic obstructive pulmonary disease.

^b^ED: emergency department.

^c^S_P_O_2_: peripheral capillary oxygen saturation.

**Table 5:** The top 3 association rules generated for the third example patient.

Rank, rule, and item on the rule’s left-hand side		Interpretation of the item	Interventions linked to the item
**Rank 1: The patient had between 24 and 49 COPD^a^ diagnoses in the past year AND the patient had >11 nebulizer medication prescriptions in the past year AND the patient is Black or an African American→ the patient will probably have at least one severe COPD exacerbation in the following 12 months**			
	The patient had between 24 and 49 COPD diagnoses in the past year	Frequently receiving COPD diagnoses indicates poor control of the disease.	Provide education on managing COPD and more frequent follow-ups Ensure use of appropriate COPD medications Consider influenza shot, pneumonia vaccination, or smoking cessation Assess the need for pulmonary rehabilitation or home care
	The patient had >11 nebulizer medication prescriptions in the past year	Using many medications for COPD with a nebulizer indicates an ineffective regimen, poor treatment adherence, or poor control of the disease. Using nebulizer medications could be a sign of having a mild exacerbation or more severe COPD.	Simplify COPD medications to once-a-day formulations or combination medications Address concerns for adverse interactions between medications Provide education on the correct use of COPD medications or inhalers Consider strategies to improve medication adherence such as providing reminders for taking medications in time Medication reconciliation review by a physician or a pharmacist
	The patient is a Black or an African American	Poor respiratory outcomes and low quality of life are more prevalent in Black and African American patients.	Ensure that the patient has needed resources and access to care Assess the need for social work or home care
**Rank 2: The patient’s last ED^b^ visit related to COPD occurred no less than 27.2 days ago and no more than 94.3 days ago AND the patient’s COPD medication prescriptions in the past year included between 13 and 16 distinct medications AND the patient’s last outpatient visit on COPD occurred no less than 82.4 days ago and no more than 327.6 days ago AND the patient’s maximum percentage of neutrophils in the past year was >76.5%** **→** **the patient will probably have at least one severe COPD exacerbation in the following 12 months**			
	The patient’s last ED visit related to COPD occurred no less than 27.2 days ago and no more than 94.3 days ago	Having a recent ED visit related to COPD shows a need for better control of the disease.	Provide education on managing COPD and more frequent follow-ups Ensure use of appropriate COPD medications Consider influenza shot, pneumonia vaccination, or smoking cessation Assess the need for pulmonary rehabilitation or home care Ensure that the patient has a primary care provider or is referred to a specialist
	The patient’s COPD medication prescriptions in the past year included between 13 and 16 distinct medications	Using many COPD medications can indicate an ineffective regimen, poor treatment adherence, or poor control of the disease.	Simplify COPD medications to once-a-day formulations or combination medications Address concerns for adverse interactions between medications Provide education on the correct use of COPD medications or inhalers Consider strategies to improve medication adherence such as using a pill organizer or providing reminders for taking medications in time Medication reconciliation review by a physician or a pharmacist
	The patient’s last outpatient visit on COPD occurred no less than 82.4 days ago and no more than 327.6 days ago	If the patient’s last outpatient visit on COPD was for acute problems with COPD, it could indicate poor control of the disease and a need for additional support to control COPD.	Provide education on managing COPD and resources for care Ensure use of appropriate COPD medications Assess the need for home care
	The patient’s maximum percentage of neutrophils in the past year was >76.5%	Having a large percentage of neutrophils can indicate infections or distress.	Evaluate the respiratory system, for example, using radiographic imaging Consider doing diagnostic tests such as viral panel, sputum culture, or procalcitonin Evaluate other potential morbidities such as cardiovascular disease with an electrocardiogram, echocardiography, or laboratory tests such as brain natriuretic peptide or D-dimer
**Rank 3:** **The patient had between 8 and 19 diagnoses of acute COPD exacerbation in the past year AND the relative decline of the patient’s BMI in the past year was >0.44% AND the patient’s total length of inpatient stays in the past year was >0.6 day** **→** **the patient will probably have at least one severe COPD exacerbation in the following 12 months**			
	The patient had between 8 and 19 diagnoses of acute COPD exacerbation in the past year	Frequently having acute COPD exacerbations shows a need for better control of the disease.	Provide education on managing COPD and more frequent follow-ups Ensure use of appropriate COPD medications Consider influenza shot, pneumonia vaccination, or smoking cessation Assess the need for pulmonary rehabilitation or home care Ensure that the patient has a primary care provider or is referred to a specialist
	The relative decline of the patient’s BMI in the past year was >0.44%	Having an unintentional weight loss can indicate comorbidities or other complications, such as malnutrition or metabolic syndrome.	Optimize nutritional status to address low BMI Provide dietary education and advise appropriate exercise
	The patient’s total length of inpatient stays in the past year was >0.6 day	Having a long inpatient stay can indicate that the patient has a more severe disease or comorbidities. Having frequent inpatient stays shows a need for better control of the disease.	Ensure that the patient has a primary care provider Assess the need for home care or referral to a skilled nursing facility Provide education on managing COPD and resources for care Ensure use of appropriate COPD medications

^a^COPD: chronic obstructive pulmonary disease.

^b^ED: emergency department.

### Performance of the Automatic Explanation Method

The automatic explanation method was evaluated using the test set. Our method explained the predictions for 97.1% (100/103) of the patients with COPD who were correctly predicted by our model to have severe COPD exacerbations in the following 12 months. For each such patient, our method gave an average of 13,880.19 (SD 18,700.60) explanations covering 39.80 (SD 11.98) distinct actionable items, a median of 4474 explanations, and a median of 41 distinct actionable items covered by the explanations. Each explanation corresponds to an association rule.

**Figure 2:**
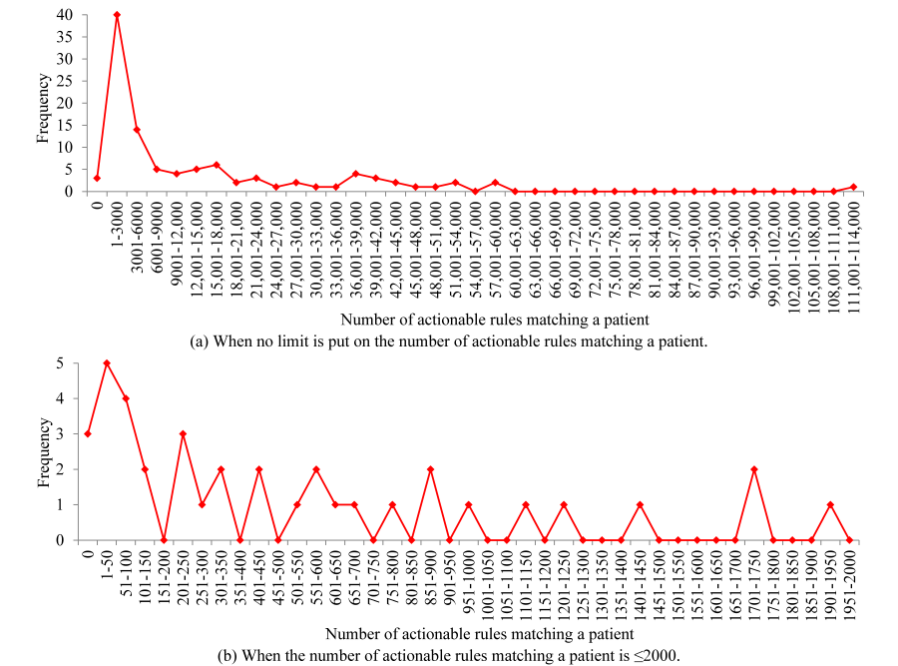
The distribution of the number of actionable rules matching a patient who was correctly predicted by our model to have ≥1 severe chronic obstructive pulmonary disease exacerbation in the following 12 months.

For the patients with COPD who were correctly predicted by our model to have severe COPD exacerbations in the following 12 months, Figure 2 shows the distribution of the number of actionable rules matching a patient. This distribution is highly skewed toward the left with a long tail. As the number of actionable rules matching a patient increases, the frequency of cases in the corresponding equal-width bucket tends to rapidly decrease in a nonmonotonic way. The largest number of actionable rules matching a patient is rather large (111,062). Nevertheless, only 1 patient matches so many rules.

**Figure 3:**
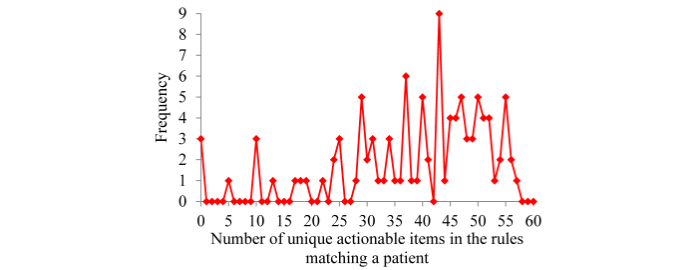
The distribution of the number of unique actionable items in the rules matching a patient who was correctly predicted by our model to have ≥1 severe chronic obstructive pulmonary disease exacerbation in the following 12 months.

For the patients with COPD who were correctly predicted by our model to have severe COPD exacerbations in the following 12 months, Figure 3 shows the distribution of the number of unique actionable items in the rules matching a patient. The largest number of unique actionable items in the rules matching a patient is 57, which is much smaller than the largest number of actionable rules matching a patient. As shown in Tables 3-5, the same intervention could be linked to ≥1 distinct actionable item in the rules matching a patient.

Our automatic explanation method explained the predictions for 73.6% (134/182) of the patients with COPD who had ≥1 severe COPD exacerbation in the following 12 months.

## Discussion

### Principal Findings

Our automatic explanation method generalizes well in predicting severe COPD exacerbations. Our method explained the predictions for 97.1% (100/103) of the patients with COPD who were correctly predicted by our model to have severe COPD exacerbations in the following 12 months. This percentage is comparable with the corresponding percentages of 87.6% to 97.6% that we previously obtained to explain the predictions of asthma outcomes [54-56]. This percentage is sufficiently large to apply our automatic explanation method to routine clinical use for COPD management. After further improving the performance of our model for predicting severe COPD exacerbations and our automatic explanation method, we hope our model can be used in conjunction with our automatic explanation method to provide decision support for allocating COPD care management resources and improve outcomes.

Our automatic explanation method explained the predictions for 73.6% (134/182) of the patients with COPD who had ≥1 severe COPD exacerbation in the following 12 months. This percentage is <97.1% (100/103), the success rate at which our method explained the predictions for the patients with COPD whom our model correctly predicted to have severe COPD exacerbations in the following 12 months. This seems likely to be because of the correlation between the prediction results of our model and the association rules. Among the patients whom our model correctly predicted to have severe COPD exacerbations in the following 12 months, many seem to be easy cases for using association rules to explain the outcomes. Among the patients who had severe COPD exacerbations but were incorrectly predicted by our model to have no severe COPD exacerbation in the following 12 months, many seem to be difficult cases for any model to correctly predict or explain the outcomes.

### Related Work

Several years ago, we designed our automatic explanation method to handle relatively balanced data and demonstrated our method for predicting the diagnosis of type 2 diabetes [58]. Later, other researchers demonstrated our method on several other clinical predictive modeling tasks, such as predicting lung transplantation or mortality in patients with cystic fibrosis [66] and predicting cardiac mortality in patients with cancer [67]. Recently, we extended our automatic explanation method so it can also handle imbalanced data, where one value of the outcome variable appears much less often than another. We demonstrated our extended method for predicting hospital encounters for asthma in patients with asthma in 3 health care systems separately [54-56]. Imbalanced data also appear in the case of predicting severe COPD exacerbations, which is the use case of this paper.

As discussed in the reviews [68,69], other researchers have developed a variety of methods to automatically explain the predictions made by machine learning models. Many of these methods lower the model performance or work only for a specific machine learning algorithm. Most of these methods provide explanations that are not of rule types. More importantly, none of these methods can automatically suggest tailored interventions, which is desired in many clinical applications. In comparison, our automatic explanation method has four properties that make it particularly suitable for providing clinical decision support: (1) it provides rule-type explanations, which are easier to understand than other kinds of explanations; (2) it works for any machine learning model on tabular data; (3) it does not lower model performance; and (4) it is the only automatic explanation method that can automatically suggest tailored interventions.

Rudin et al [70], Ribeiro et al [71], Rasouli et al [72], Pastor and Baralis [73], Guidotti et al [74], and Panigutti et al [75] used rules to automatically explain machine learning predictions. These rules are not known before the time of prediction, making it impossible to use them to automatically suggest tailored interventions at the time of prediction. Except for the case of Pastor and Baralis [73], these rules are not association rules. In comparison, our automatic explanation method mines association rules before the time of prediction and uses them to automatically suggest tailored interventions at the time of prediction.

### Limitations

This study has 5 limitations that are worth addressing in future work.

First, this study used data from a single health care system. It is worth assessing our automatic explanation method’s performance in explaining the predictions of severe COPD exacerbations in other health care systems.

Second, this study focuses on the prediction of one outcome—whether a patient with COPD will have ≥1 severe COPD exacerbation in the following 12 months. It is worth assessing our automatic explanation method’s performance in explaining the predictions of other outcomes.

Third, our automatic explanation method currently works for explaining the predictions that traditional non–deep-learning machine learning algorithms make on tabular data. It is worth investigating the extension of our method to handle the predictions made by deep learning models on longitudinal data [76,77].

Fourth, we currently know no optimal way to present automatic explanations and automatically suggested interventions. It is worth investigating an optimal way to present this information based on a user-centered design.

Finally, researchers have assessed the impact of automatic explanations on decision-making for several other applications [78-82] before but not for care management. For the automatic explanation function for predicting severe COPD exacerbations presented in this paper, it is worth assessing the impact of showing automatic explanations and automatically suggested interventions on care management enrollment and intervention decisions.

### Conclusions

Our automatic explanation method generalizes well in predicting severe COPD exacerbations. After further improving the performance of our model for predicting severe COPD exacerbations and our automatic explanation method, we hope our model can be used in conjunction with our automatic explanation method to provide decision support for allocating COPD care management resources and improve outcomes.

## Abbreviations

COPD: chronic obstructive pulmonary disease
ED: emergency department
UWM: University of Washington Medicine
XGBoost: extreme gradient boosting

## References

[1] (2019). Disease or condition of the week - COPD. Centers for Disease Control and Prevention.

[2] Ford ES, Murphy LB, Khavjou O, Giles WH, Holt JB, Croft JB (2015). Total and state-specific medical and absenteeism costs of COPD among adults aged ≥ 18 years in the United States for 2010 and projections through 2020. Chest.

[3] (2020). 2020 Gold Reports. Global Initiative for Chronic Obstructive Lung Disease - GOLD.

[4] Anzueto A, Leimer I, Kesten S (2009). Impact of frequency of COPD exacerbations on pulmonary function, health status and clinical outcomes. Int J Chron Obstruct Pulmon Dis.

[5] Connors Jr AF, Dawson NV, Thomas C, Harrell Jr FE, Desbiens N, Fulkerson WJ, Kussin P, Bellamy P, Goldman L, Knaus WA (1996). Outcomes following acute exacerbation of severe chronic obstructive lung disease. The SUPPORT investigators (Study to Understand Prognoses and Preferences for Outcomes and Risks of Treatments). Am J Respir Crit Care Med.

[6] Viglio S, Iadarola P, Lupi A, Trisolini R, Tinelli C, Balbi B, Grassi V, Worlitzsch D, Döring G, Meloni F, Meyer KC, Dowson L, Hill SL, Stockley RA, Luisetti M (2000). MEKC of desmosine and isodesmosine in urine of chronic destructive lung disease patients. Eur Respir J.

[7] Kanner RE, Anthonisen NR, Connett JE, Lung Health Study Research Group (2001). Lower respiratory illnesses promote FEV(1) decline in current smokers but not ex-smokers with mild chronic obstructive pulmonary disease: results from the lung health study. Am J Respir Crit Care Med.

[8] Spencer S, Jones PW, GLOBE Study Group (2003). Time course of recovery of health status following an infective exacerbation of chronic bronchitis. Thorax.

[9] Spencer S, Calverley PM, Burge PS, Jones PW, ISOLDE (Inhaled Steroids in Obstructive Lung Disease) Study Group (2001). Health status deterioration in patients with chronic obstructive pulmonary disease. Am J Respir Crit Care Med.

[10] Blanchette CM, Dalal AA, Mapel D (2012). Changes in COPD demographics and costs over 20 years. J Med Econ.

[11] Johnston J, Longman J, Ewald D, King J, Das S, Passey M (2020). Study of potentially preventable hospitalisations (PPH) for chronic conditions: what proportion are preventable and what factors are associated with preventable PPH?. BMJ Open.

[12] Curry N, Billings J, Darin B, Dixon J, Williams M, Wennberg D (2005). Predictive risk project literature review. King's Fund, London.

[13] Mays GP, Claxton G, White J (2004). Managed care rebound? Recent changes in health plans' cost containment strategies. Health Aff (Millwood).

[14] Bandurska E, Damps-Konstańska I, Popowski P, Jędrzejczyk T, Janowiak P, Świętnicka K, Zarzeczna-Baran M, Jassem E (2017). Impact of integrated care model (ICM) on direct medical costs in management of advanced chronic obstructive pulmonary disease (COPD). Med Sci Monit.

[15] Rice KL, Dewan N, Bloomfield HE, Grill J, Schult TM, Nelson DB, Kumari S, Thomas M, Geist LJ, Beaner C, Caldwell M, Niewoehner DE (2010). Disease management program for chronic obstructive pulmonary disease: a randomized controlled trial. Am J Respir Crit Care Med.

[16] Axelrod RC, Vogel D (2003). Predictive modeling in health plans. Dis Manag Health Outcomes.

[17] Zeng S, Arjomandi M, Tong Y, Liao ZC, Luo G (2022). Developing a machine learning model to predict severe chronic obstructive pulmonary disease exacerbations: retrospective cohort study. J Med Internet Res.

[18] Annavarapu S, Goldfarb S, Gelb M, Moretz C, Renda A, Kaila S (2018). Development and validation of a predictive model to identify patients at risk of severe COPD exacerbations using administrative claims data. Int J Chron Obstruct Pulmon Dis.

[19] Tavakoli H, Chen W, Sin DD, FitzGerald JM, Sadatsafavi M (2020). Predicting severe chronic obstructive pulmonary disease exacerbations. Developing a population surveillance approach with administrative data. Ann Am Thorac Soc.

[20] Samp JC, Joo MJ, Schumock GT, Calip GS, Pickard AS, Lee TA (2018). Predicting acute exacerbations in chronic obstructive pulmonary disease. J Manag Care Spec Pharm.

[21] Thomsen M, Ingebrigtsen TS, Marott JL, Dahl M, Lange P, Vestbo J, Nordestgaard BG (2013). Inflammatory biomarkers and exacerbations in chronic obstructive pulmonary disease. J Am Med Assoc.

[22] Orchard P, Agakova A, Pinnock H, Burton CD, Sarran C, Agakov F, McKinstry B (2018). Improving prediction of risk of hospital admission in chronic obstructive pulmonary disease: application of machine learning to telemonitoring data. J Med Internet Res.

[23] Suetomo M, Kawayama T, Kinoshita T, Takenaka S, Matsuoka M, Matsunaga K, Hoshino T (2014). COPD assessment tests scores are associated with exacerbated chronic obstructive pulmonary disease in Japanese patients. Respir Investig.

[24] Lee SD, Huang MS, Kang J, Lin CH, Park MJ, Oh YM, Kwon N, Jones PW, Sajkov D, Investigators of the Predictive Ability of CAT in Acute Exacerbations of COPD (PACE) Study (2014). The COPD assessment test (CAT) assists prediction of COPD exacerbations in high-risk patients. Respir Med.

[25] Faganello MM, Tanni SE, Sanchez FF, Pelegrino NR, Lucheta PA, Godoy I (2010). BODE index and GOLD staging as predictors of 1-year exacerbation risk in chronic obstructive pulmonary disease. Am J Med Sci.

[26] Alcázar B, García-Polo C, Herrejón A, Ruiz LA, de Miguel J, Ros JA, García-Sidro P, Conde GT, López-Campos JL, Martínez C, Costán J, Bonnin M, Mayoralas S, Miravitlles M (2012). Factors associated with hospital admission for exacerbation of chronic obstructive pulmonary disease. Arch Bronconeumol.

[27] Bertens LC, Reitsma JB, Moons KG, van Mourik Y, Lammers JW, Broekhuizen BD, Hoes AW, Rutten FH (2013). Development and validation of a model to predict the risk of exacerbations in chronic obstructive pulmonary disease. Int J Chron Obstruct Pulmon Dis.

[28] Miravitlles M, Guerrero T, Mayordomo C, Sánchez-Agudo L, Nicolau F, Segú JL (2000). Factors associated with increased risk of exacerbation and hospital admission in a cohort of ambulatory COPD patients: a multiple logistic regression analysis. The EOLO Study Group. Respiration.

[29] Make BJ, Eriksson G, Calverley PM, Jenkins CR, Postma DS, Peterson S, Östlund O, Anzueto A (2015). A score to predict short-term risk of COPD exacerbations (SCOPEX). Int J Chron Obstruct Pulmon Dis.

[30] Montserrat-Capdevila J, Godoy P, Marsal JR, Barbé F (2015). Predictive model of hospital admission for COPD exacerbation. Respir Care.

[31] Kerkhof M, Freeman D, Jones R, Chisholm A, Price DB, Respiratory Effectiveness Group (2015). Predicting frequent COPD exacerbations using primary care data. Int J Chron Obstruct Pulmon Dis.

[32] Chen X, Wang Q, Hu Y, Zhang L, Xiong W, Xu Y, Yu J, Wang Y (2020). A nomogram for predicting severe exacerbations in stable COPD patients. Int J Chron Obstruct Pulmon Dis.

[33] Yii AC, Loh CH, Tiew PY, Xu H, Taha AA, Koh J, Tan J, Lapperre TS, Anzueto A, Tee AK (2019). A clinical prediction model for hospitalized COPD exacerbations based on "treatable traits". Int J Chron Obstruct Pulmon Dis.

[34] Adibi A, Sin DD, Safari A, Johnson KM, Aaron SD, FitzGerald JM, Sadatsafavi M (2020). The Acute COPD Exacerbation Prediction Tool (ACCEPT): a modelling study. Lancet Respir Med.

[35] Stanford RH, Nag A, Mapel DW, Lee TA, Rosiello R, Vekeman F, Gauthier-Loiselle M, Duh MS, Merrigan JF, Schatz M (2016). Validation of a new risk measure for chronic obstructive pulmonary disease exacerbation using health insurance claims data. Ann Am Thorac Soc.

[36] Stanford RH, Nag A, Mapel DW, Lee TA, Rosiello R, Schatz M, Vekeman F, Gauthier-Loiselle M, Merrigan JF, Duh MS (2018). Claims-based risk model for first severe COPD exacerbation. Am J Manag Care.

[37] Stanford RH, Lau MS, Li Y, Stemkowski S (2019). External validation of a COPD risk measure in a commercial and Medicare population: the COPD treatment ratio. J Manag Care Spec Pharm.

[38] Stanford RH, Korrer S, Brekke L, Reinsch T, Bengtson LG (2020). Validation and assessment of the COPD treatment ratio as a predictor of severe exacerbations. Chronic Obstr Pulm Dis.

[39] Jones RC, Donaldson GC, Chavannes NH, Kida K, Dickson-Spillmann M, Harding S, Wedzicha JA, Price D, Hyland ME (2009). Derivation and validation of a composite index of severity in chronic obstructive pulmonary disease: the DOSE Index. Am J Respir Crit Care Med.

[40] Jones RC, Price D, Chavannes NH, Lee AJ, Hyland ME, Ställberg B, Lisspers K, Sundh J, van der Molen T, Tsiligianni I, UNLOCK Group of the IPCRG (2016). Multi-component assessment of chronic obstructive pulmonary disease: an evaluation of the ADO and DOSE indices and the global obstructive lung disease categories in international primary care data sets. NPJ Prim Care Respir Med.

[41] Fan VS, Curtis JR, Tu SP, McDonell MB, Fihn SD, Ambulatory Care Quality Improvement Project Investigators (2002). Using quality of life to predict hospitalization and mortality in patients with obstructive lung diseases. Chest.

[42] Moy ML, Teylan M, Danilack VA, Gagnon DR, Garshick E (2014). An index of daily step count and systemic inflammation predicts clinical outcomes in chronic obstructive pulmonary disease. Ann Am Thorac Soc.

[43] Briggs A, Spencer M, Wang H, Mannino D, Sin DD (2008). Development and validation of a prognostic index for health outcomes in chronic obstructive pulmonary disease. Arch Intern Med.

[44] Lange P, Marott JL, Vestbo J, Olsen KR, Ingebrigtsen TS, Dahl M, Nordestgaard BG (2012). Prediction of the clinical course of chronic obstructive pulmonary disease, using the new GOLD classification: a study of the general population. Am J Respir Crit Care Med.

[45] Abascal-Bolado B, Novotny PJ, Sloan JA, Karpman C, Dulohery MM, Benzo RP (2015). Forecasting COPD hospitalization in the clinic: optimizing the chronic respiratory questionnaire. Int J Chron Obstruct Pulmon Dis.

[46] Blanco-Aparicio M, Vázquez I, Pita-Fernández S, Pértega-Diaz S, Verea-Hernando H (2013). Utility of brief questionnaires of health-related quality of life (Airways Questionnaire 20 and Clinical COPD Questionnaire) to predict exacerbations in patients with asthma and COPD. Health Qual Life Outcomes.

[47] Yoo JW, Hong Y, Seo JB, Chae EJ, Ra SW, Lee JH, Kim EK, Baek S, Kim TH, Kim WJ, Lee JH, Lee SM, Lee S, Lim SY, Shin TR, Yoon HI, Sheen SS, Lee JS, Huh JW, Oh YM, Lee SD (2011). Comparison of clinico-physiologic and CT imaging risk factors for COPD exacerbation. J Korean Med Sci.

[48] Niewoehner DE, Lokhnygina Y, Rice K, Kuschner WG, Sharafkhaneh A, Sarosi GA, Krumpe P, Pieper K, Kesten S (2007). Risk indexes for exacerbations and hospitalizations due to COPD. Chest.

[49] Austin PC, Stanbrook MB, Anderson GM, Newman A, Gershon AS (2012). Comparative ability of comorbidity classification methods for administrative data to predict outcomes in patients with chronic obstructive pulmonary disease. Ann Epidemiol.

[50] Marin JM, Carrizo SJ, Casanova C, Martinez-Camblor P, Soriano JB, Agusti AG, Celli BR (2009). Prediction of risk of COPD exacerbations by the BODE index. Respir Med.

[51] Ställberg B, Lisspers K, Larsson K, Janson C, Müller M, Łuczko M, Bjerregaard B, Bacher G, Holzhauer B, Goyal P, Johansson G (2021). Predicting hospitalization due to COPD exacerbations in Swedish primary care patients using machine learning - based on the ARCTIC study. Int J Chron Obstruct Pulmon Dis.

[52] Chen T, Guestrin C (2016). XGBoost: a scalable tree boosting system. Proceedings of the ACM SIGKDD International Conference on Knowledge Discovery and Data Mining.

[53] (2018). U.S. healthcare leaders expect widespread adoption of artificial intelligence by 2023. Intel.

[54] Luo G, Johnson MD, Nkoy FL, He S, Stone BL (2020). Automatically explaining machine learning prediction results on asthma hospital visits in patients with asthma: secondary analysis. JMIR Med Inform.

[55] Tong Y, Messinger AI, Luo G (2020). Testing the generalizability of an automated method for explaining machine learning predictions on asthma patients' asthma hospital visits to an academic healthcare system. IEEE Access.

[56] Luo G, Nau CL, Crawford WW, Schatz M, Zeiger RS, Koebnick C (2021). Generalizability of an automatic explanation method for machine learning prediction results on asthma-related hospital visits in patients with asthma: quantitative analysis. J Med Internet Res.

[57] Zhang X, Luo G (2021). Ranking rule-based automatic explanations for machine learning predictions on asthma hospital encounters in patients with asthma: retrospective cohort study. JMIR Med Inform.

[58] Luo G (2016). Automatically explaining machine learning prediction results: a demonstration on type 2 diabetes risk prediction. Health Inf Sci Syst.

[59] (2012). NQF #1891 Hospital 30-day, all-cause, risk-standardized readmission rate (RSRR) following chronic obstructive pulmonary disease (COPD) hospitalization. National Quality Forum.

[60] Cooke CR, Joo MJ, Anderson SM, Lee TA, Udris EM, Johnson E, Au DH (2011). The validity of using ICD-9 codes and pharmacy records to identify patients with chronic obstructive pulmonary disease. BMC Health Serv Res.

[61] Nguyen HQ, Chu L, Amy Liu LI, Lee JS, Suh D, Korotzer B, Yuen G, Desai S, Coleman KJ, Xiang AH, Gould MK (2014). Associations between physical activity and 30-day readmission risk in chronic obstructive pulmonary disease. Ann Am Thorac Soc.

[62] Lindenauer PK, Grosso LM, Wang C, Wang Y, Krishnan JA, Lee TA, Au DH, Mularski RA, Bernheim SM, Drye EE (2013). Development, validation, and results of a risk-standardized measure of hospital 30-day mortality for patients with exacerbation of chronic obstructive pulmonary disease. J Hosp Med.

[63] Liu B, Hsu W, Ma Y (1998). Integrating classification and association rule mining. Proceedings of the 4th International Conference on Knowledge Discovery and Data Mining.

[64] Thabtah FA (2007). A review of associative classification mining. Knowledge Eng Review.

[65] Fayyad UM, Irani KB (1993). Multi-interval discretization of continuous-valued attributes for classification learning. Proceedings of the 13th International Joint Conference on Artificial Intelligence.

[66] Alaa AM, van der Schaar M (2018). Prognostication and risk factors for cystic fibrosis via automated machine learning. Sci Rep.

[67] Alaa AM, van der Schaar M (2018). AutoPrognosis: automated clinical prognostic modeling via Bayesian optimization with structured kernel learning. Proceedings of the 35th International Conference on Machine Learning.

[68] Guidotti R, Monreale A, Ruggieri S, Turini F, Giannotti F, Pedreschi D (2019). A survey of methods for explaining black box models. ACM Comput Surv.

[69] Payrovnaziri SN, Chen Z, Rengifo-Moreno P, Miller T, Bian J, Chen JH, Liu X, He Z (2020). Explainable artificial intelligence models using real-world electronic health record data: a systematic scoping review. J Am Med Inform Assoc.

[70] Rudin C, Shaposhnik Y (2019). Globally-consistent rule-based summary-explanations for machine learning models: application to credit-risk evaluation. Proceedings of INFORMS 11th Conference on Information Systems and Technology.

[71] Ribeiro MT, Singh S, Guestrin C (2018). Anchors: high-precision model-agnostic explanations. Proceedings of the 32nd AAAI Conference on Artificial Intelligence.

[72] Rasouli P, Yu IC (2020). EXPLAN: explaining black-box classifiers using adaptive neighborhood generation. Proceedings of the 2020 International Joint Conference on Neural Networks.

[73] Pastor E, Baralis E (2019). Explaining black box models by means of local rules. Proceedings of the 34th ACM/SIGAPP Symposium on Applied Computing.

[74] Guidotti R, Monreale A, Ruggieri S, Pedreschi D, Turini F, Giannotti F (2018). Local rule-based explanations of black box decision systems. arXiv.

[75] Panigutti C, Perotti A, Pedreschi D (2020). Doctor XAI: an ontology-based approach to black-box sequential data classification explanations. Proceedings of the Conference on Fairness, Accountability, and Transparency.

[76] Luo G, Stone BL, Koebnick C, He S, Au DH, Sheng X, Murtaugh MA, Sward KA, Schatz M, Zeiger RS, Davidson GH, Nkoy FL (2019). Using temporal features to provide data-driven clinical early warnings for chronic obstructive pulmonary disease and asthma care management: protocol for a secondary analysis. JMIR Res Protoc.

[77] Luo G (2019). A roadmap for semi-automatically extracting predictive and clinically meaningful temporal features from medical data for predictive modeling. Glob Transit.

[78] Weerts HJ, van Ipenburg W, Pechenizkiy M (2019). A human-grounded evaluation of SHAP for alert processing. arXiv.

[79] Stites MC, Nyre-Yu M, Moss B, Smutz C, Smith MR (2021). Sage advice? The impacts of explanations for machine learning models on human decision-making in spam detection. Proceedings of the Second International Conference on Artificial Intelligence in HCI.

[80] Lai V, Tan C (2019). On human predictions with explanations and predictions of machine learning models: a case study on deception detection. Proceedings of the Conference on Fairness, Accountability, and Transparency.

[81] Lundberg SM, Nair B, Vavilala MS, Horibe M, Eisses MJ, Adams T, Liston DE, Low DK, Newman SF, Kim J, Lee SI (2018). Explainable machine-learning predictions for the prevention of hypoxaemia during surgery. Nat Biomed Eng.

[82] Jesus SM, Belém C, Balayan V, Bento J, Saleiro P, Bizarro P, Gama J (2021). How can I choose an explainer? An application-grounded evaluation of post-hoc explanations. Proceedings of the 2021 ACM Conference on Fairness, Accountability, and Transparency.

